# Noise analysis of Grover and phase estimation algorithms implemented as quantum singular value transformations for a small number of noisy qubits

**DOI:** 10.1038/s41598-023-47246-x

**Published:** 2023-11-17

**Authors:** Muhammad Abdullah Ijaz, Muhammad Faryad

**Affiliations:** Department of Physics, Lahore University of Management Sciences, Lahore, 54792 Pakistan

**Keywords:** Quantum information, Quantum simulation

## Abstract

The quantum singular value transformation (QSVT) algorithm is a general framework to implement most of the known algorithms and provides a way forward for designing new algorithms. In the present work, the impact of noise on the QSVT algorithm is examined for bit flip, phase flip, bit-phase flip, and depolarizing noise models for a small number of qubits. The small number of noisy qubits approximates the currently available noisy quantum computers. For simulation results, the QSVT implementation of the Grover search and quantum phase estimation (QPE) algorithms is considered. These algorithms are among the basic quantum algorithms and form the building blocks of various applications of quantum algorithms. The results showed that the QSVT implementation of the Grover search and QPE algorithms has a consistently worse dependence upon noise than the original implementation for all four noise models. The probability of success of the Grover algorithm and phase measured by the QPE algorithm were found to exponentially depend upon the error probability in the noisy channels but only linearly dependent on the number of qubits.

## Introduction

Grover search and quantum phase estimation (QPE) are two major quantum algorithms that act as building blocks for many other quantum algorithms^1^. Both of these algorithms provide distinct speed-up over their classical counterparts. Recently, a unifying quantum algorithm, namely, the quantum singular value transformation (QSVT) algorithm, was discovered that generalizes many known algorithms, including Grover search and QPE^2^.

The QSVT framework was essentially anticipated earlier as quantum signal processing and block encoding^3–6^ before its consolidation^2^ with the specializations to Grover search, phase estimation, Hamiltonian simulation, and HHL following later^7^. The QSVT allows for the implementation of arbitrary polynomials^2,8,9^, which need only be sufficiently well-behaved. Moreover, since QSVT uses singular value decomposition, non-square and non-unitary operations can also be implemented in the QSVT circuit. The advantages of the QSVT regime are undeniable, as we can use it to implement a wide range of simple algorithms like the Grover search and complex algorithms like the feedback-dependent phase estimation. Not only do the QSVT implementations match the complexity advantage of the non-QSVT Grover search and phase estimation algorithm in the limit of large qubits, but the search problem can also be adjusted to avoid the convergence problem, and the phase estimation in the QSVT framework implements ${\hat{U}}_{p}^{2^{j}}$ more reliably^7^.

In the limit of large qubits, both the original quantum algorithms and their QSVT generalizations provide similar algorithmic complexity. However, in the limit of a small number of qubits, the overhead of the QSVT algorithm demands a higher gate count for basic quantum algorithms like Grover search and QPE than the original implementations of these algorithms. This is important because of the small and noisy current quantum computers that are very sensitive to the number of gates in a given circuit^10^. Therefore, we set out to investigate the impact of various noise models on implementing Grover and QPE algorithms using QSVT in the limit of the small number of qubits. For this purpose, the salient features relevant to our work of the QSVT, Grover, and QPE algorithms are discussed in Sec. The Algorithms. The simulation results with various noise models are presented in Sec. Results and Discussion, and concluding remarks are presented in Sec. Conclusions.

All the operators are decorated with a hat, vectors are represented with uppercase Greek alphabets, and the quantum states are represented using the usual Dirac notations.

## The algorithms

### Quantum singular value transformation

The basis of the QSVT algorithm is the singular value transformation, which can decompose any arbitrary matrix ${\hat{A}}$ as

1 $$\begin{aligned} {\hat{A}} = \sum _{k=1}^{r} \sigma _k \vert u_k\rangle \langle v_k\vert , \end{aligned}$$

where the singular values $\sigma _k$ are non-negative and real. Furthermore, ${\hat{A}}$ has the left and right vector spaces that are spanned by $\{\vert u_k\rangle \}$ and $\{\vert v_k\rangle \}$, respectively. This description of ${\hat{A}}$ allows us to write any polynomial of this matrix as a function of its singular values $\sigma _k$. This property is a necessary condition for block encoding ${\hat{A}}$ as

$$\begin{aligned} {\hat{U}} = \begin{bmatrix} {\hat{A}} &{} \sqrt{I - {\hat{A}}^{2}}\\ \sqrt{I - {\hat{A}}^{2}} &{} -{\hat{A}} \end{bmatrix}\,, \end{aligned}$$

where

2 $$\begin{aligned} \sqrt{I-A^2}=\sum _{k=1}^{r}\sqrt{1- \sigma _k^2} \vert u_k\rangle \langle v_k\vert . \end{aligned}$$

Using this process we can encode ${\hat{A}}$ within a subspace of ${\hat{U}}$ accessible by the projection operator ${{\hat{P}} = \sum _{k} \vert u_k\rangle \langle u_k\vert }$ and ${{\hat{P}}}^{'} = \sum _{k} \vert v_k\rangle \langle v_k\vert$, such that ${\hat{A}} = {{\hat{P}}}^{'}{\hat{U}} {{\hat{P}}}$^2^. We must construct projection-controlled phase operators to utilize this block encoding for the QSVT algorithms, which will substitute the rotation operator in the signal processing framework. These operators take the form ${{{\hat{P}}}^{'}}_{\phi _i}$ and ${{{\hat{P}}}}_{\phi _j}$, which induces a phase to the ancilla qubit when our system is in the subspace where ${\hat{A}}$ is encoded. Substituting these unitary ${\hat{U}}$ and projection-controlled phase operators in the quantum signal processing algorithm allows for the construction of unitaries^6^

$$\begin{aligned} \begin{aligned}{}&{\hat{U}}_{\Phi } = {{{\hat{P}}}^{'}}_{\phi _1} {\hat{U}} \left[ \prod _{k=1}^{(d-1)/2} {{{\hat{P}}}}_{\phi _{2k}} {\hat{U}}^{\dag } {{{\hat{P}}}^{'}}_{\phi _{2k+1}} {\hat{U}}\right] ,{} & {} d\in \{1,3,5,...\},\\&{\hat{U}}_{\Phi } = \prod _{k=1}^{d/2} {{{\hat{P}}}}_{\phi _{2k-1}} {\hat{U}}^{\dag } {{{\hat{P}}}^{'}}_{\phi _{2k+1}} {\hat{U}} ,{} & {} d\in \{2,4,6,...\} , \end{aligned} \end{aligned}$$

which can implement any polynomial ${Poly}^{(SV)}({\hat{A}})$ on the domain of ${ \sigma _k \in [-1,1]}$ with the rotation parameters $\Phi = (\phi _1,\phi _2,...,\phi _d)$ and has the properties^11^ :

1. $${Poly}({\hat{A}}) \le d$$,
2. ${Poly}({\hat{A}})$ has parity d mod(2),
3. $${\mid {Poly}({\hat{A}}) \mid }^{2} + {\mid {Poly}(\sqrt{I - {\hat{A}}^{2}} ) \mid }^{2} = 1.$$

Armed with these operators, we need only determine the polynomial that approximates the problem along with the rotation parameters $\Phi = (\phi _1,\phi _2,...,\phi _d)$ to construct ${\hat{U}}_{\Phi }$^9,11^. For example, the HHL algorithm can be implemented in the QSVT framework to solve a set of linear equations efficiently^12^ where the polynomial used is a well-behaved approximation of $f(x) = 1/x$ with $\Phi$ determined using algorithms such as Remez-type exchange^8,13^.

Within the scope of this paper, we will only focus on the Grover search and phase estimation algorithm with the rotation parameters

3 $$\begin{aligned} \Phi = \left( (1-d)\frac{\pi }{2},\frac{\pi }{2},...,\frac{\pi }{2}\right) , \end{aligned}$$

which allow for the construction of any $d\text{th}$ order Chebyshev polynomial required for the Grover search and QPE^6,7^.

### Grover search algorithm

**Figure 1:**
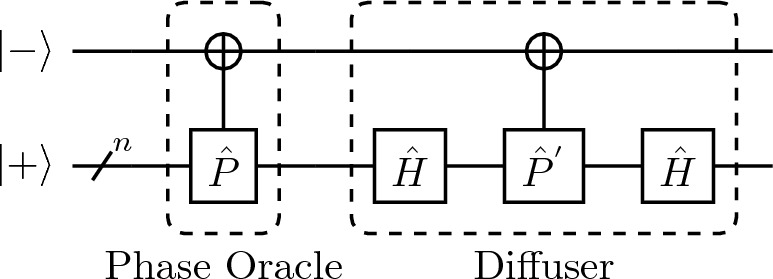
The non-QSVT quantum circuit for Grover search algorithm, composed of the phase oracle and diffuser, which amplify the state using the phase kicked back onto the ancilla qubit.

The Grover search algorithm searches for a marked state within an unstructured search space. This is achieved by the amplification of the probability amplitude of the marked state over *r* iteration of the Grover Oracle. The traditional structure of the Grover oracle contains two operators, the phase oracle, ${\hat{U}}_{g}$ defined as

4 $$\begin{aligned} {\hat{U}}_{g} \vert j\rangle = (-1)^{\delta _{jm}} \vert j\rangle \, \end{aligned}$$

which kicks back a negative phase onto the marked state $\vert m\rangle$, and the diffuser, that amplifies this state’s probability amplitude. Since we want to amplify the states $\vert m\rangle$ from an initial state of uniform superposition $\vert \psi _{0}\rangle = \vert +\rangle ={\hat{H}}^{\otimes n} \vert 0\rangle$, the state evolves such that ${\hat{U}}_{\Phi } \vert \psi _{0}\rangle \rightarrow {} \vert m\rangle$, where ${\hat{H}}^{\otimes n}$ is the Hadamard transform. The Grover algorithm is a specific case of the amplitude amplification algorithm, and if the system does not initially contain the marked state, the algorithm always fails. The circuit diagram of the non-QSVT Grover is given in Fig. 1. A key factor in this circuit is that the ancilla qubit is initialized in an eigenstate of ${\hat{X}}$ which reduces gate complexity for the phase oracle.

The QSVT equivalent of the Grover search algorithm using the phase oracle defined in Eq. (4) can be written as the polynomial that satisfies the condition

5 $$\begin{aligned} Poly(c_{m}) = \langle m\vert {\hat{U}}_{\Phi } \vert \psi _{0}\rangle , \end{aligned}$$

where $c_{m}$ is the probability amplitude of $\vert m\rangle$ in $\vert \psi _{0}\rangle$ state. Hence, the polynomial has odd parity and the upper-bound is $Poly(1/\sqrt{2^n}) \le 1$. Therefore, we can use the ${\hat{U}}_{\Phi }$ defined for odd parity.

**Figure 2:**
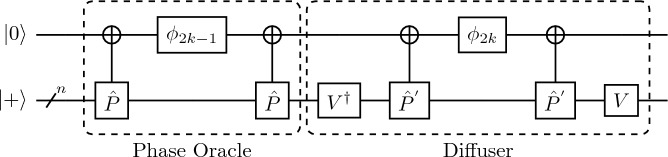
The QSVT quantum circuit for Grover search algorithm, composed of the phase rotation gate $\phi$, which takes rotation parameters as input, the unitary ${\hat{V}}= {\hat{H}}^{\otimes n}$ operators and the $C_{{{\hat{P}}}}NOT$ and $C_{{{\hat{P}}}'}NOT$ gates that target the ancilla qubit.

The QSVT implementation of the Grover search algorithm is shown in Fig. 2 with the phase oracle and diffuser highlighted. The first part of the QSVT Grover iterate, with the phase gate sandwiched between the $C_{{{\hat{P}}}}NOT$ gates is ${{{\hat{P}}}}_{\phi _j}$ which is equivalent to the oracle ${\hat{U}}_{g}$ as it attaches a negative phase to the marked state. At the same time, the rest acts as the diffuser and reflects the state around the uniform superposition state $\vert \psi _{0}\rangle$. The amplitude amplification process for the QSVT Grover search is also identical to the non-QSVT, except gate expense for the phase induction is much higher. This circuit uses ${\mathscr{O}}(2^{n/2} (1/\delta ))$ queries for a single marked state to the projection-controlled phase operators for both the left and right subspace and the preparation unitary ${\hat{V}}= {\hat{H}}^{\otimes n}$ operator. The $\delta$ is the error tolerance and leads to the probability of success $\ge 1 - \delta$^7^. For the Grover search the projection operators are defined as ${{\hat{P}}} = {\hat{I}} - \vert m\rangle \langle m\vert$ and ${{\hat{P}}}^{'} = {\hat{I}} - \vert 0\rangle \langle 0\vert$.

Hence, with the combination of these gates in the order shown in Fig. 2, we see the reproduction of the non-QSVT Grover search algorithm. This implies that the QSVT Grover oracle can be resolved using geometric arguments and requires *r* queries for the maximum amplitude of the marked state because of its sinusoidal amplitude amplification relation with iterations. Let us note that the non-QSVT Grover search influenced the working principle of signal processing used in QSVT through amplitude amplification. That is why, we can draw parallels between the algorithms and identify the non-QSVT oracle and diffuser within the unique circuitry of the QSVT framework.

### Quantum phase estimation

The quantum phase estimation algorithm was developed as an application of the Quantum Fourier Transform (QFT) and allows us to find the eigenvalue of the operator ${\hat{U}}_{p},$ which can be represented as

6 $$\begin{aligned} {\hat{U}}_{p}\vert u\rangle = e^{2 \pi i\theta }\vert u\rangle . \end{aligned}$$

**Figure 3:**
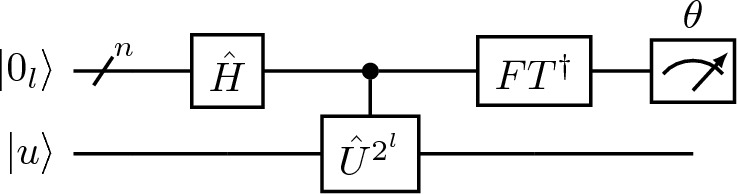
The $l\text{th}$ section of the non-QSVT quantum circuit for phase estimation algorithm.

The algorithm outputs the phase $\theta$ and assumes that we have a preparation oracle that constructs the state $\vert u\rangle$ and can implement ${\hat{U}}_{p}^j$ with high accuracy. The non-QSVT phase estimation algorithm shown in Fig. 3 uses *n* qubit to output $\theta$, where the $j\text{th}$ bit of $\theta$ is encoded into the $(n-j)\text{th}$ qubit using the inverse QFT.

The QSVT implementation of the QPE algorithm is based on Kitaev’s iterative algorithm and approximates the phase introduced by a unitary ${\hat{U}}_{p}$, using a semi-classical feedback process^14,15^. The Kitaev method employs the less significant bits of $\theta$ to estimate the next significant bit; hence, it requires the phase of each bit to be encoded into the system state before the next bit can be approximated. This encoding cannot be achieved using the inverse QFT but can be represented as a polynomial, which enables the construction of the QSVT QPE algorithm^7,16^.

Since the QSVT implementation of the QPE is iterative, the $l\text{th}$ loop approximates the $\theta _{l}$ by encoding the less significant bits. For this we can expand the induced phase as $\theta = 0.\theta _{1}\theta _{2}...\theta _{l}...\theta _{n}$ and phase encoded after the $l\text{th}$ iteration as $\theta ^{'} = 0.\theta _{l+1},\theta _{l+2}...\theta _{n}$. The sign function is the polynomial required for estimating and encoding the $l\text{th}$ bit. However, since it is discontinuous, we instead use a symmetrized polynomial approximate, with even parity and an upper bound of magnitude 1^7^.

**Figure 4:**
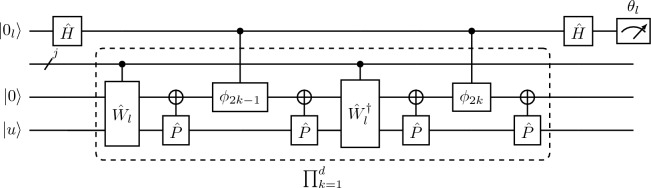
The $l\text{th}$ section of the QSVT quantum circuit for phase estimation algorithm, where $\phi$ is a phase rotation gate which takes the rotation parameters as argument and the $C_{{{\hat{P}}}}NOT$ and $C_{{{\hat{P}}}'}NOT$ gates target the ancilla qubit.

The QSVT circuit corresponding to this polynomial is shown in Fig. 4, where *d* determines the accuracy of each iteration. This block traverses from $l = n-1,n-2.. 0$ where ${\hat{W}}_{l}(\theta ^{'})$ takes the less significant bits as input and can be represented as

$$\begin{aligned} {\hat{W}}_{j}(\theta ^{'}) = \frac{1}{2}\begin{bmatrix} {\hat{I}} + e^{-2\pi i \theta ^{'}} {\hat{U}}_{p}^{2^{j}} &{} {\hat{I}} - e^{-2\pi i \theta ^{'}} {\hat{U}}_{p}^{2^{j}}\\ {\hat{I}} - e^{-2\pi i \theta ^{'}} {\hat{U}}_{p}^{2^{j}} &{} {\hat{I}} + e^{-2\pi i \theta ^{'}} {\hat{U}}_{p}^{2^{j}} \end{bmatrix} . \end{aligned}$$

**Figure 5:**
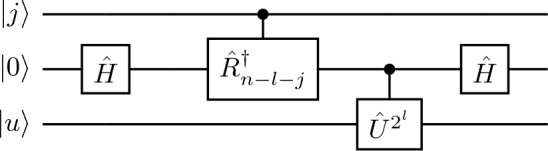
The expanded form of the ${\hat{W}}_{l-1}$ is given above, where ${\hat{R}}_\vartheta$ is a controlled phase rotation, such that ${\hat{R}}_\vartheta = {\hat{R}}_{z}(2 \pi /2^{\vartheta })$ and is applied for $j \in \{ 0,1,...,n-l-2 \}$.

The circuit for the operator ${\hat{W}}_{l}(\theta ^{'})$ is shown in Fig. 5, and makes ${\mathscr{O}}(n-l)$ queries to the controlled rotation gates each of which is controlled by the projection operators. Furthermore, the $l\text{th}$ iteration makes ${\mathscr{O}}(n log(n/\delta ))$ queries to the operator ${\hat{W}}_j$, and the projection controlled operators $C_{{{\hat{P}}}'}NOT$ where ${{\hat{P}}} = {{\hat{P}}}^{'} = \vert 0\rangle \langle 0\vert \otimes {\hat{I}}$. On the last iteration the circuit outputs $\theta ^{'}$, with the error $|\theta - \theta ^{'}| < 1/2^n$ and probability of success $\ge 1-\delta$, where $\delta$ is the error tolerance. Although the inverse QFT is not applied in the QSVT QPE circuit, its block-encoded equivalent emerges after working out the circuit for $d = 1$ and separating all the unitaries and the rotation gates.

## Results and discussion

In this work, we used the noise model representing the simplest noise processes in a single qubit. The QSVT and non-QSVT algorithms are compared for the bit flip, phase flip, bit-phase flip, and the depolarizing noise channel. The evolution of the state under the noisy channel can be characterized as,

7 $$\begin{aligned} \varepsilon ({\hat{\rho }}) = \sum _{k=1} {\hat{E}}_{k} {\hat{\rho }}{\hat{E}}^{\dagger }_{k}\,, \end{aligned}$$

where the Kraus operators ${\hat{E}}_{k}$ depend on the channel such that $\sum {\hat{E}}^{\dagger }_{k} {\hat{E}}_{k} = {\hat{I}}$. Operators corresponding to the noise models are provided in Table 1.

For all the numerical results in the paper, the algorithms were decomposed into the form of the single qubit basic gates set $\{{\hat{I}}, {\hat{X}}, \sqrt{{\hat{X}}}, {\hat{R}}z, CNOT\}$ before the noise models were introduced into the gates. For the single qubit gates, the noise channel was applied right after each gate operation. For the two-qubit *CNOT* gate, the noise channels were applied to both the control and target qubits independent of each other^17^. The results in this section were produced after the algorithm was simulated for 10, 000 shots and measured in the computational basis as a function of the probability of error *p*.

**Table 1:** The Kraus operators corresponding to the different noisy channels used for the noise analysis in Eq. (7).

Channel	Kraus Operators	
Depolarizing	$\sqrt{1-3p/4}{\hat{I}}$, $\sqrt{p/4}{\hat{\sigma }}_{i}$	
Bit flip	$\sqrt{1-p}{\hat{I}}$, $\sqrt{p}{\hat{X}}$	
Phase flip	$\sqrt{1-p}{\hat{I}}$, $\sqrt{p}{\hat{Z}}$	
Bit-phase flip	$\sqrt{1-p}{\hat{I}}$, $\sqrt{p}{\hat{Y}}$	

The ${\hat{\sigma }}_{i}$ signifies that all Pauli gates are used for the depolarizing channel.

### Grover search algorithm

**Figure 6:**
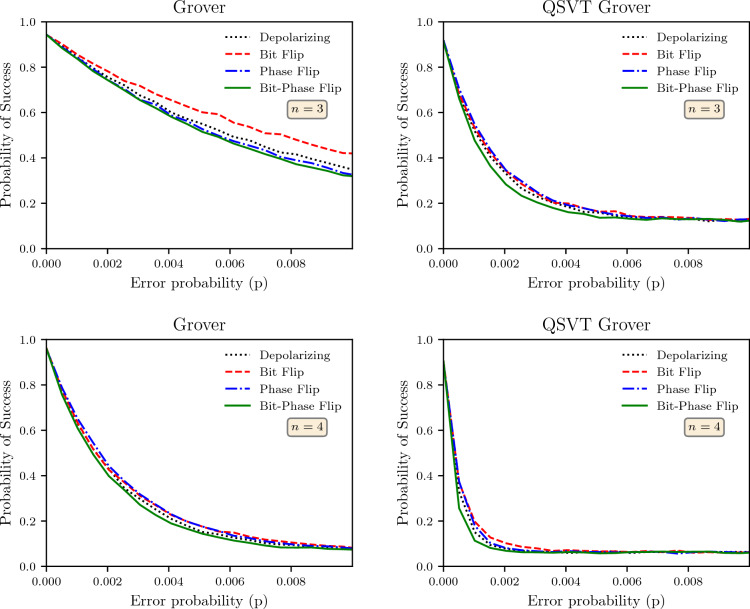
The probability of success for various noise models for non-QSVT and QSVT Grover search algorithms when the number of working qubits was 3 or 4. The probability of success was computed by running the algorithm for 10, 000 shots at each value of *p*.

The Grover search algorithms implemented for this report have the marked state $\vert m\rangle = \vert 1\rangle ^{\otimes n}$ and the corresponding projection-controlled operator ${{\hat{P}}} = {\hat{I}} - \vert 11..1\rangle \langle 11...1\vert$. The algorithm’s efficiency is analyzed using the probability of marked state after *r* iterations of the Grover oracle and is referred to as the probability of success. This probability of success for the non-QSVT and QSVT Grover search is plotted in Fig. 6 against the probability of error *p* for the search space of dimensions $n = \{3,4\}$.

**Figure 7:**
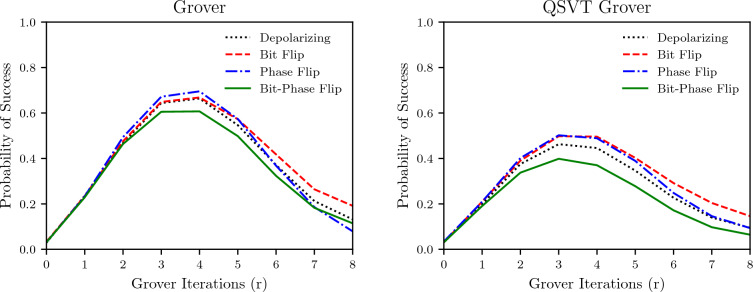
The probability of success against the number of iterations of the non-QSVT and QSVT Grover oracle for different noise channels with fixed probability of error $p=0.0003$ and number of qubits $n=5$.

The plots show that the probability of success inherent to the QSVT Grover is higher than that of the non-QSVT Grover for any given value of *p*. Across all results, for high value *p*, the probability of success approaches a $1/2^n$, which is the coefficient of the marked state in the maximally mixed state $I/2^n$. Therefore, the noise drives the system’s output to a maximally mixed state. However, this approach to maximally mixed state comes for smaller value of *p* for QSVT algorithm than the non-QSVT implementation. The results also show that with an increase in the number of qubits, the probability of success reduces. Furthermore, all the the noise channels have approximately the same dependence except the bit-phase flip channel that has consistently the worst impact on the probability of success.

For the same probability of error *p*, we can also see that the QSVT Grover search algorithm performs worse than the non-QSVT algorithm, and these results can be generalized to higher dimensions. As discussed above, the QSVT implementation contains the $C_{{{\hat{P}}}}NOT_{\phi }$ and $V^{\dagger }C_{{{\hat{P}}}^{'}}NOT_{\phi }V$ which are equivalent to the phase oracle and diffuser of the non-QSVT algorithm respectively. The results in Fig. 6 show that the the probability of success depends exponentially upon the probability of error of the noise channel. This exponential dependence is stronger for the QSVT than the original algorithm.

Since the Grover algorithm merely rotates the marked state in a space spanned by the desired output state and the state orthogonal to it, the output is a periodic function of the number of iterations. This error accumulation against the number of iterations is plotted in Fig. 7 for $n = 5$ and probability of error $p = 0.0003$. The optimum number of iterations of the oracle for the search space $n=5$ and a single marked state is $r=4$. Hence, for a noiseless oracle, we would have amplitude maximized at $r =4$, which matches the result for non-QSVT Grover, but for QSVT Grover, the maximum amplitude of marked state is achieved at $r=3$ showing that the noise impact is so high that the information gained by going to $r=4$ using the QSVT algorithm is outpaced by the error in this iteration. The probability of success for the non-QSVT is also significantly higher than the QSVT algorithm. These results also match the result from Fig. 6; the QSVT performs worse than the non-QSVT Grover algorithm, and both have the worst performance for the bit-phase flip noise channel.

**Table 2:** The number of gates and depth of the circuit for non-QSVT and QSVT Grover algorithms.

n	Non-QSVT			QSVT		
	Single qubit gates	*CNOT* gates	Depth	Single qubit gates	*CNOT* gates	Depth
2	29	12	28	95	60	119
3	54	28	58	259	196	367
4	248	144	297	999	648	1328
5	633	552	927	2160	2024	3403

The higher noise impact on the QSVT algorithm is due to a higher number of gates than the non-QSVT algorithm for the same number of working qubits. This dependence can be seen in Table 2 where we can see that the gate count for the QSVT is higher than the non-QSVT and the number of extra gates increases with the dimension of the search space *n*. This is also true for the depth of the circuit which quantifies the highest number of sequential gate operations on a qubit in the system. Another notable features is a particularly high count of CNOT gates for the QSVT algorithm. This is because QSVT algorithm performs many operations as controlled operations, which gives rise very high number of CNOT gates.

**Figure 8:**
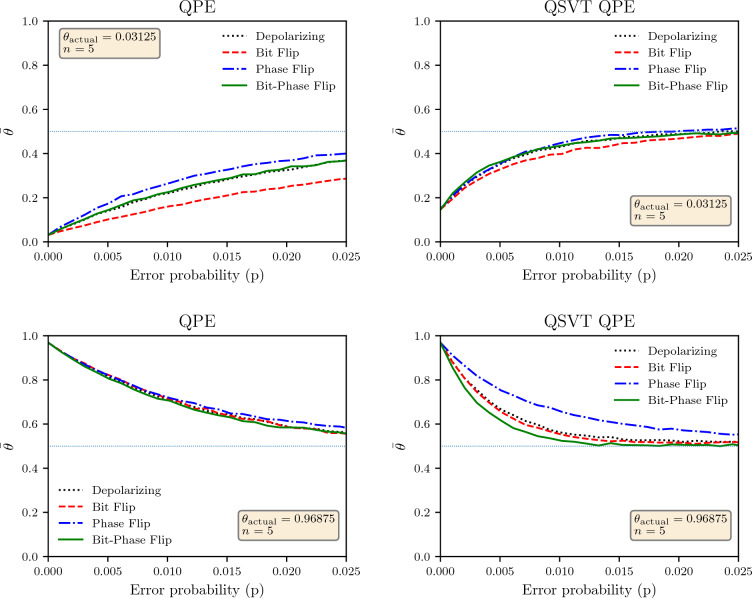
Average value of $\theta ^{'}$ against the probability of error *p*, for non-QSVT and QSVT QPE algorithm for two values of $\theta$. The dotted line shows the average value of $\theta ^{`}$ for a maximally mixed state.

### Phase estimation algorithm

For the noise analysis of the phase estimation algorithm, we used the expectation value of the measured phase $\theta ^{'}$ when the actual phase $\theta$ was introduced using the unitary ${\hat{U}}$. For all results, we have used $n=5$ qubits and have used $d =2$ for the QSVT algorithm. The average value of observed $\theta ^{'}$ was used as our metric for comparing the two implementations.

The simulation results for the average value of $\theta ^{'}$ is shown in Fig. 8 against the probability of error *p* for $\theta =\theta _{l}= 0.00001$ (in binary) and $\theta =\theta _{h}= 0.11111$ (in binary) as two extremal values. For $\theta _{h}$ simulation of non-QSVT QPE, the channels have similar noise impact for all the channels, but for $\theta _{l}$, the algorithm is most robust against the bit flip channels and least for the phase flip channel. For $\theta =\theta _{h}$, the QSVT QPE performs significantly better against the phase flip channel but is more susceptible to the bit-phase flip channel. Across all results, we see that for a low value of *p*, the average value of $\theta ^{'}$ is close to the actual value but eventually approaches $\sim 0.5$ when *p* increases because the output state approaches the maximally mixed state. However, the approach to the maximally mixed state is more rapid for the QSVT than the non-QSVT implementation of the QPE. Just like the Grover, the expected value of the output phase seem to exponentially depend upon the error probability of the noise channels both for the QSVT and the original formulation, and this exponential dependence is stronger for the QSVT than the original formulation. Furthermore, a comparison with the Grover algorithm shows that the QPE is much more robust to noise than the Grover algorithm.

**Figure 9:**
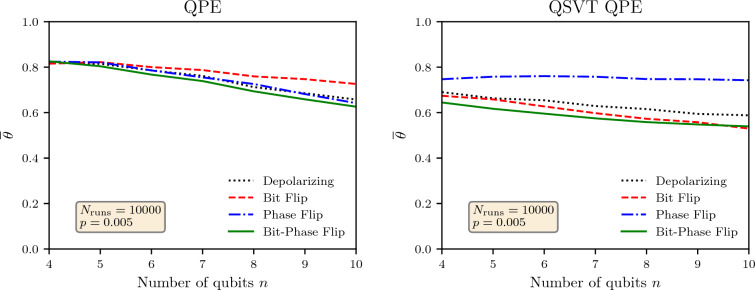
Average value of $\theta ^{'}$ against the number of working qubits *n* with probability of error $p = 0.005$ for non-QSVT and QSVT QPE algorithms. The actual phase in the unitary operator $U_p$ was set at $1-2^{-n}$.

To understand the scaling of noise with the number of qubits, the expected value of $\theta ^{'}$ for different numbers of qubits *n* is shown in Fig. 9. The actual value of the phase was set as $1-2^{-n}$ since this was the maximum possible value of the phase for given number of qubits. We needed a consistent value of actual phase for comparing the output across different number of qubits. The expected value of $\theta ^{'}$ for $n = 5$ match the results shown in Fig. 8. The results in Fig. 9 shows that the output state approaches maximally mixed state as number of qubits increase even for a fixed error probability $p=0.005$. Though the rate of approach slightly depends upon the noise channel except for the phase-fllip noise for the QSVT QPE. The scan of the results show that the dependence of the expected value of $\theta$ is almost linearly dependent upon the number of qubits, as opposed to exponential dependence upon the error probability of the noise channels.

**Table 3:** Number of gates for non-QSVT and QSVT QPE algorithms.

n	Non-QSVT			QSVT		
	Single qubit gates	*CNOT* Gates	Depth	Single qubit gates	*CNOT* gates	Depth
2	28	6	21	94	34	97
3	36	14	33	159	64	171
4	52	25	44	235	102	254
5	68	35	55	319	148	351

The number of single and double-qubit gates is given in Table 3 along with the depth of the circuit. The table shows that an increase in *n* not only increases the number of gates, but also the depth of the circuit. The increase in gates and depth increase the error in the output of the algorithm. Furthermore, the table clearly shows a significantly higher number of gates and higher depth for the QSVT algorithm than the original implementation. This explains the worse perofrmance of the QSVT algorithm than the original formulation.

## Conclusions

We simulated the implementation of Grover search and quantum phase estimation (QPE) algorithms using original and quantum singular value transformation (QSVT) algorithms for a small number of working qubits relevant to noisy quantum computers. We decomposed the QSVT algorithm and showed how the non-QSVT equivalent of those circuits emerged. It was shown that the probability of success for Grover and the expected phase measured from the QPE showed stronger dependence upon the error probability for QSVT implementation than the original algorithm. This stronger dependence of the QSVT algorithm is due to higher number of one- and two-qubit gates required than the original formulation. Furthermore, the increase in the depth of the circuit with the increase in the number of qubits makes the output of the QPE algorithm approach maximally mixed state when the number of qubits are increased even at fixed error probability of the noise channels. Similar results were seen for all four noise channels considered in this work. The probability of success of the Grover algorithm and phase measured by the QPE algorithm were found to exponentially depend upon the error probability in the noisy channels but only linearly dependent on the number of qubits. Let us note that more investigations are needed to understand the dependence upon the error probability and the number of qubits.

A comparison of results of QPE and Grover algorithm shows that the Grover algorithm’s dependence upon the noise is stronger than the QPE both for the original formulation and for the QSVT implementation. This is because of the higher number of gates and higher depth for the Grover algorithm than the QPE both for the original and for the QSVT implementation. The consistently worse performance for the QSVT implementation across four different types of noise models shows that the QSVT algorithm’s advantage is limited on today’s noisy quantum computers^10^.

## Data Availability

All data generated or analysed during this study are included in this published article.
